# Identification of immunity- and ferroptosis-related signature genes as potential design targets for mRNA vaccines in AML patients

**DOI:** 10.18632/aging.206068

**Published:** 2024-08-29

**Authors:** Chaojie Wang, Liping Lv, Ping Ma, Yangyang Zhang, Mingyuan Li, Jiang Deng, Yanyu Zhang

**Affiliations:** 1 Institute of Health Service and Transfusion Medicine, Beijing 100850, P.R. China; 2 Beijing Key Laboratory of Blood Safety and Supply Technologies, Beijing 100850, P.R. China; 3 College of Biotechnology, Tianjin University of Science and Technology, Tianjin 300457, P.R. China

**Keywords:** acute myeloid leukemia, tumor mutational burden, immune landscape, mRNA vaccine design

## Abstract

Immune-associated ferroptosis plays an important role in the progression of acute myeloid leukemia (AML); however, the targets that play key roles in this process are currently unknown. This limits the development of mRNA vaccines based on immune-associated ferroptosis for clinical therapeutic applications. In this study, based on the rich data resources of the TCGA-LAML cohort, we analyzed the tumor mutational burden (TMB), gene mutation status, and associations between immune and ferroptosis genes to reveal the disease characteristics of AML patients. To gain a deeper understanding of differentially expressed genes, we applied the Limma package for differential expression analysis and integrated data sources such as ImmPort Shared Data and FerrDb V2. Moreover, we established gene modules related to TMB according to weighted gene coexpression network analysis (WGCNA) and explored the functions of these modules in AML and their relationships with TMB. We focused on the top 30 most frequent genes through a detailed survey of missense mutations and single nucleotide polymorphisms (SNPs) and selected potentially critical gene targets for subsequent analysis. Based on the expression of these genes, we successfully subgrouped AML patients and found that the subgroups associated with TMB (C1 and C2) exhibited significant differences in survival. The differences in the tumor microenvironment and immune cells between C1 and C2 patients were investigated with the ESTIMATE and MCP-counter algorithms. A predictive model of TMB-related genes (TMBRGs) was constructed, and the validity of the model was demonstrated by categorizing patients into high-risk and low-risk groups. The differences in survival between the high-risk patients and high-TMB patients were further investigated, and potential vaccine targets were identified via immune cell-level analysis. The identification of immunity- and ferroptosis-associated signature genes is an independent predictor of survival in AML patients and provides new information on immunotherapy for AML.

## INTRODUCTION

In acute myeloid leukemia (AML), hematopoietic stem cells exhibit abnormal proliferation, survival, and differentiation [1–3]. Conventional therapies for AML include chemotherapy [4, 5] and stem cell transplantation [6, 7]. Chemotherapy destroys leukemia cells by using cytotoxic drugs to inhibit abnormal proliferation [8]. Although chemotherapy has achieved significant therapeutic effects in some patients, it is often accompanied by a series of side effects, such as immunosuppression, nausea, and hair loss, which have significant impacts on patient quality of life [9]. Conversely, the application of stem cell transplantation has been limited due to the difficulty of donor matching and the possibility of rejection and other complications after transplantation [10, 11]. Although conventional therapies play a role in AML treatment, their efficacy is still unsatisfactory. Therefore, the search for more innovative and individualized treatment modalities has become urgent for improving outcomes and survival rates in patients with AML.

With the rise of individualized therapy [12] and precision medicine, immunotherapy, an emerging field of cancer treatment, has provided a new treatment pathway for AML patients [13, 14]. Over the past few years, mRNA vaccine technology has developed rapidly [15] and has shown great potential in the field of cancer immunotherapy [16, 17]. By delivering synthetic mRNAs encoding specific antigens to the body, mRNA vaccines stimulate a patient’s immune system to produce targeted antibodies or T-cell responses, thus facilitating a precise fight against cancer cells [18]. Compared with traditional vaccines, mRNA vaccines can be designed and produced more rapidly and flexibly and can respond rapidly to the mutation of pathogens or heterogeneous tumors [19]. Moreover, mRNA vaccines are highly individualized and can be customized according to a patient’s genetic background and disease characteristics, improving the precision of treatment. This personalized treatment approach provides a new way to treat AML and other tumor types [20] and is expected to constitute a breakthrough in the field of treatment. However, nevertheless the process of preparing mRNA vaccines is becoming increasingly mature, it is not yet possible to develop a mature mRNA vaccine for AML; primarily because no suitable mRNA vaccine target has been identified [21] that can effectively trigger a rapid and effective antitumor immune response in patients [13].

In ferroptosis, reactive oxygen species (ROS) are upregulated in response to iron, which is different from other programmed cell death pathways, such as apoptosis, necrosis, and autophagy [22]. In ferroptosis, lipid peroxidation of unsaturated fatty acids on the cell membrane is catalyzed by Fe2+ or ester oxygenase, resulting in cell death by lipid peroxidation. Ferroptosis is intricately linked to the pathophysiology of cancer [23, 24], neurological diseases, and other diseases; therefore, ferroptosis has been an important focus of disease research in recent years. Several researchers have screened mRNA vaccine targets based on the mechanism of ferroptosis [25], but these targets have not yet been identified in AML-related studies. Therefore, we investigated ferroptosis as a new strategy for screening mRNA vaccine targets [26, 27].

In this context, we aimed to explore the characteristic genes related to immunization and ferroptosis as potential targets for mRNA vaccine design in AML patients. Through bioinformatic analysis, we successfully identified four characteristic genes associated with ferroptosis, providing us with the possibility of identifying new therapeutic targets. Our study aimed to fill this gap in the existing research and provide a new direction for mRNA vaccine design in AML patients by identifying ferroptosis-related genes.

## RESULTS

### Tumor-associated gene mutation landscape

**Figure 1:**
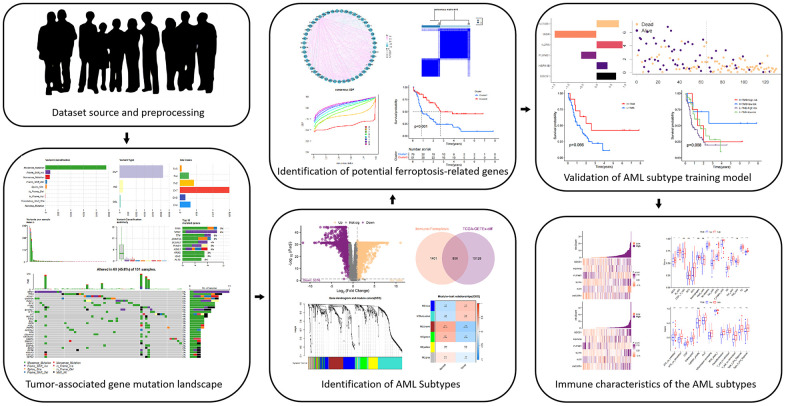
An overview of the study procedure is shown in the flow chart above.

**Figure 2:**
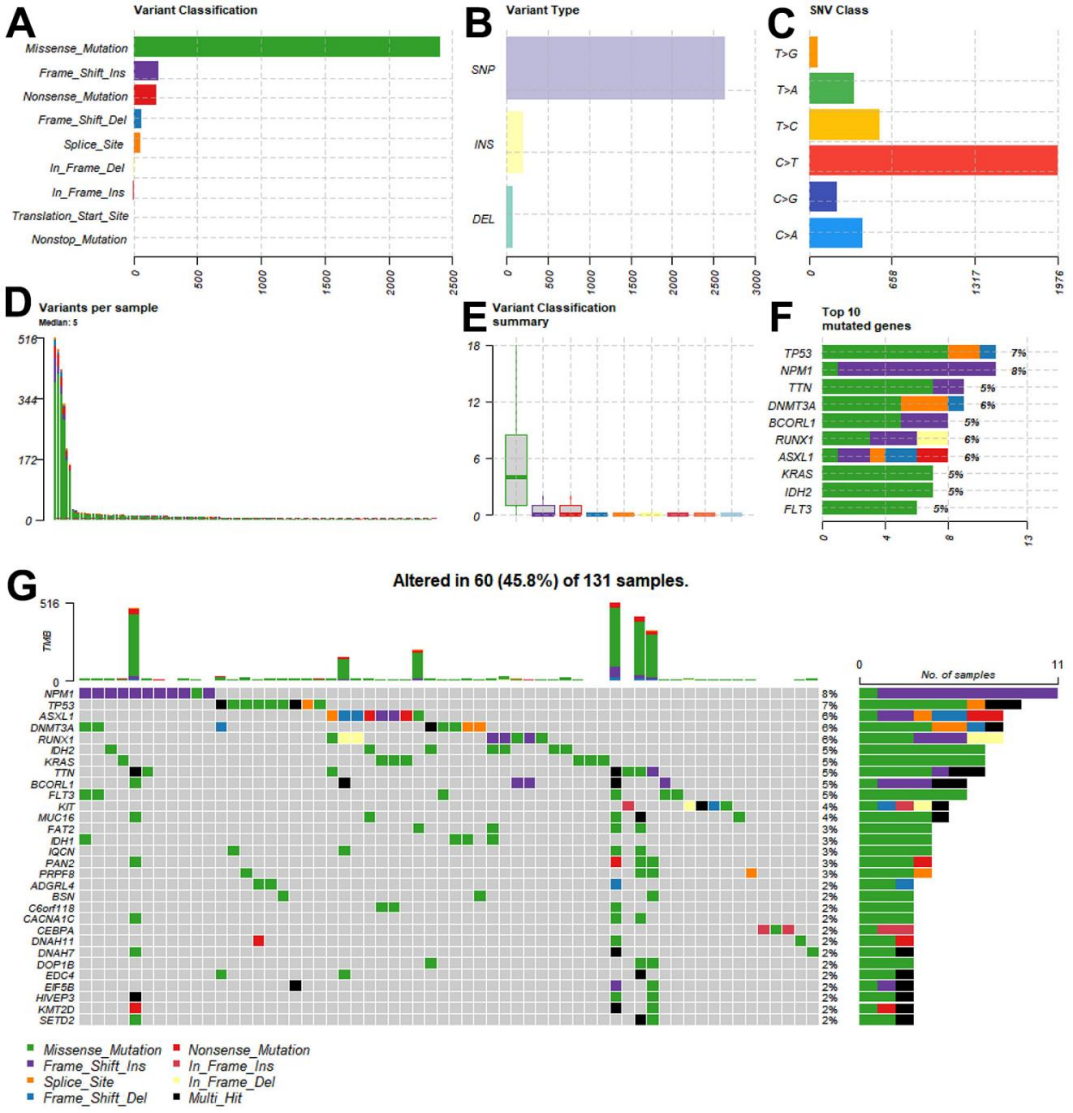
**Tumor mutational burden of patients in the TCGA-LAML cohort.** (**A**–**E**) The multiple forms of genomic mutations in the AML patients. (**A**) Variant classification. (**B**) Variant type. (**C**) SNV class. (**D**) Variants per sample. (**E**) Variant classification summary. (**F**) Mutations in the 10 most common genes. (**G**) The landscape of the genomic alterations in the top 30 genes per patient.

Figure 1 shows the flowchart of how we conducted our study. In the TCGA-LAML cohort, we first examined the mutational burden of each patient (Figure 2A–2E). Multiple variant types, including missense mutations and SNPs, were analyzed to gain a comprehensive understanding of the genetic variability characterizing the tumors. This cohort was characterized by missense mutations as the most common type of variation, while SNPs were the most common form of variation. Among the SNV classifications, the most common was C > T. We further explored the mutations in the 10 most common genes (Figure 2F), and additional detailed information about the mutations in the top 30 genes in each patient is shown in Figure 2G.

### Identification of potential ferroptosis-related genes

**Figure 3:**
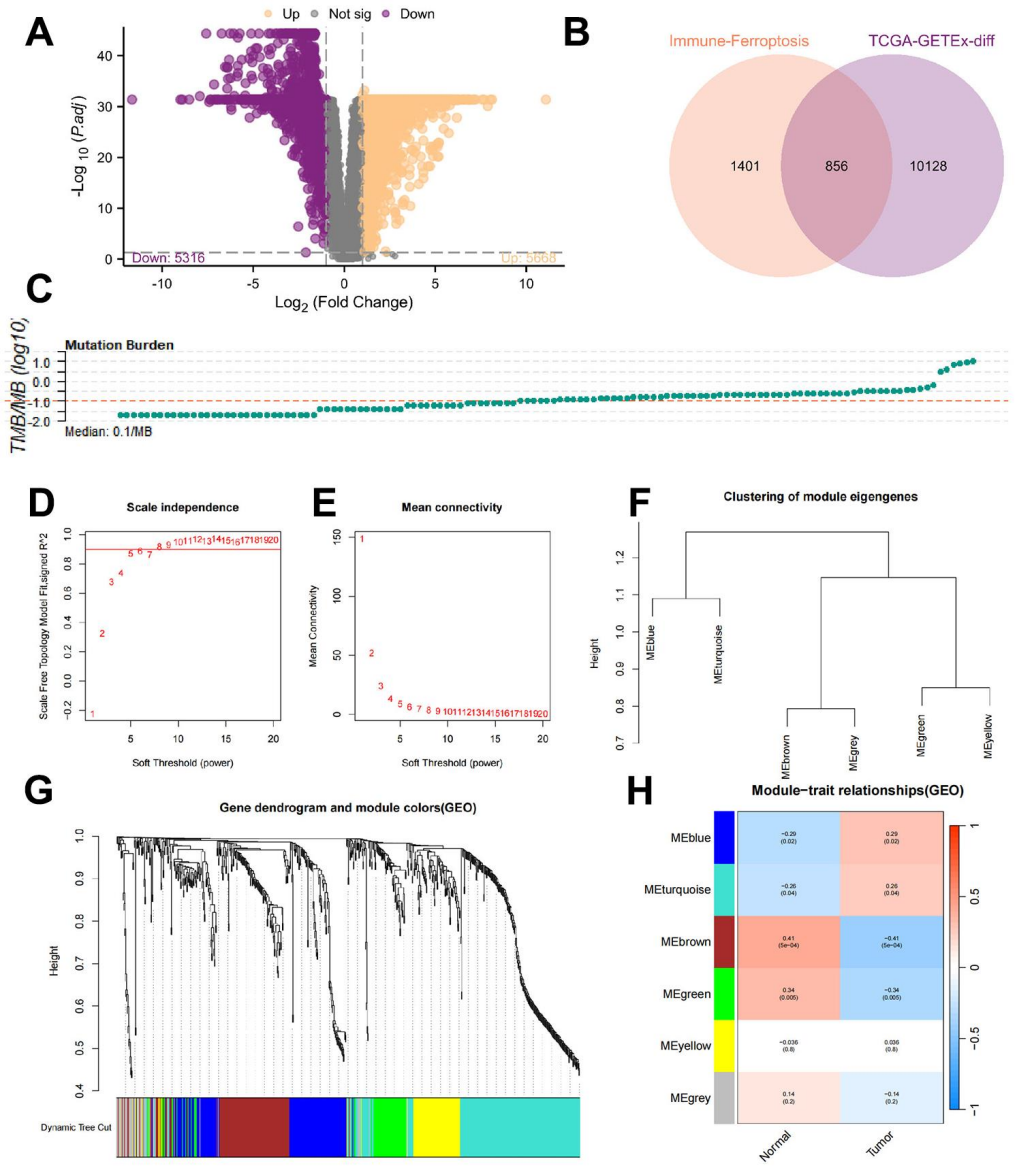
**Identification of the ferroptosis-related genes.** (**A**) Differential gene screening revealed a total of 5668 upregulated genes and 5316 downregulated genes based on the threshold. (**B**) By taking the intersection, 856 genes were identified as candidate genes for subsequent analysis. (**C**) Accumulation of TMB in each patient. (**D**, **E**) To establish scale-free networks, the soft thresholding power was set to β=6 based on scale independence and mean connectivity. (**F**, **G**) The dynamic tree cut package was used to generate a gene cluster dendrogram containing 6 coexpression models. (**H**) The coexpression models are shown in blue, turquoise, brown, green, yellow, and gray and contain 146, 218, 128, 70, 81, and 43 genes, respectively. p <0.05 was considered to indicate a significant module-trait relationship between the TMBRGs and vital status.

Due to the lack of controls for the LAML cohort in the TCGA data frame, we performed batch correction and merged the TCGA-LAML data with the GTEx data; next, we performed differential analysis based on the Limma package in R to identify DEGs. Figure 3A shows the DEG screening and a thresholding analysis identified 5668 upregulated and 5316 downregulated genes. By integrating data from ImmPort Shared Data (https://immport.org/shared/home) and FerrDb V2 (http://www.zhounan.org/ferrdb/current), a gene list of 2257 genes was compiled. The intersection of 856 genes was taken as a candidate gene for further analysis (Figure 3B).

Even though LAML development is associated with many changes in immunity and ferroptosis regulation, we do not know whether all of these genes are mutated during the tumor process, which is especially critical to vaccine function. The accumulation of TMB in each patient was then examined (Figure 3C), defining the top one-third of patients with the highest TMB as “high mutation load patients” and the bottom one-third as “low mutation load patients”. Subsequently, WGCNA was used to identify gene modules associated with TMB among immune and ferroptosis genes.

In order to establish scale-free networks, the soft thresholding power was set to β=6 as a result of scale independence and mean connectivity (Figure 3D–3E). A gene cluster dendrogram containing six coexpression models was generated using the dynamic tree cut package (Figure 3F–3G). The coexpression models are shown in blue, turquoise, brown, green, yellow, and gray and contain 146, 218, 128, 70, 81, and 43 genes, respectively. Vital status and TMBRG have a significant module-trait relationship when p <0.05 was considered significant (Figure 3H). As a result of these analyses, the 562 genes in the blue, turquoise, brown, and green modules were selected for further analysis.

### Identification of AML subtypes

**Figure 4:**
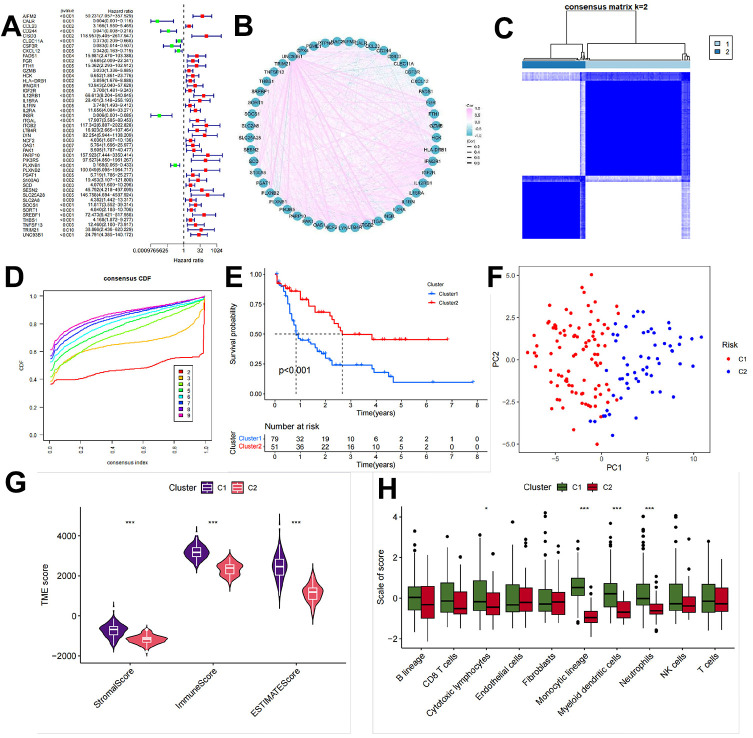
**Identification of AML subtypes.** (**A**) Based on one-way Cox regression analysis, the TMBRGs were screened, and p<0.01 was used as the threshold to filter out the prognosis-related TMBRGs. The results are shown in the form of forest plots. (**B**) To explore the correlation between the different genes, the correlation of prognosis-related genes was evaluated. (**C**, **D**) The K-means clustering algorithm was used to classify the patients into different clusters resulting in the C1 and C2 clusters. (**E**) Survival analysis showing that the prognosis of patients in the C2 group was significantly better than that of patients in the C1 group (p<0.001). (**F**, **G**) Tumor microenvironment analysis of the LAML cohort using the ESTIMATE algorithm and further exploration of the differences in the tumor microenvironments of the patients in clusters C1 and C2. (**H**) The use of the MCP-counter algorithm to further quantify the absolute abundance of 8 immune cells and 2 stromal cells in the 2 subgroups after the ESTIMATE calculation of tumor stromal and immune components was performed. These cells included T cells, CD8+ T cells, cytotoxic lymphocytes, B lineage cells, NK cells, monocytic lineage cells, myeloid lineage cells, and NK cells, as well as the absolute abundance of the tumor stromal and immune components in the 2 subgroups. Monocytic lineage cells, myeloid dendritic cells, neutrophils, endothelial cells, and fibroblasts.

Since the immune response to mRNA vaccines has different effects on different populations, we next continued to explore whether patients could be differentiated into subgroups based on TMBRGs. The TMBRGs were screened based on one-way Cox regression analysis, with p < 0.01 serving as the threshold for identifying TMBRGs associated with prognosis; the results are presented as a forest plot in Figure 4A. Genes associated with prognosis were examined to explore the correlation between the different genes (Figure 4B). Almost all genes expressed positively in response to UNC93B1. Then, based on the K-means clustering algorithm, different groups of patients were classified, namely, the C1 and C2 groups (Figure 4C, 4D). Through PCA, we found that the LAML cohort was more clearly distinguished. An analysis of survival data revealed that patients in the C2 group had a significantly better prognosis than those in the C1 group (Figure 4E, p<0.001). Predicting vaccine effectiveness requires an understanding of the tumor immune microenvironment [28]. Based on the ESTIMATE algorithm, we analyzed the tumor microenvironment in the LAML cohort and explored the differences between the C1 and C2 cohorts (Figure 4G). As a result, the C1 patients’ stromal, immune, and ESTIMATE scores were significantly higher than those of the C2 patients (all p < 0.001), C1 and C2 patients had significantly different tumor microenvironments. We further quantified the absolute abundance of 8 immune cell types and 2 stromal cell types in 2 subpopulations according to the MCP-counter algorithm after calculating the stromal and immune components of the tumors via the ESTIMATE algorithm: T cells, CD8+ T cells, cytotoxic lymphocytes, B lineage cells, NK cells, monocytic lineage cells, myeloid lineage cells, and NK cells. The cells were classified as monocytic lineage cells, myeloid dendritic cells, neutrophils, endothelial cells, or fibroblasts (Figure 4H). Patients with C1 had significantly higher levels of monocytic lineage cells, myeloid dendritic cells, and neutrophils than patients with C2, and the above results indicate that the TMBRGs were able to differentiate between the two types of patients whose tumor microenvironments were different. Notably, unlike in patients with solid tumors, in the LAML cohort, we found that the subgroup with the best prognosis had higher levels of immune cells, which may be due to the massive expansion of immature cancer cells, which are immune cells, after the onset of leukemia.

### Validation of the AML subtype training model

**Figure 5:**
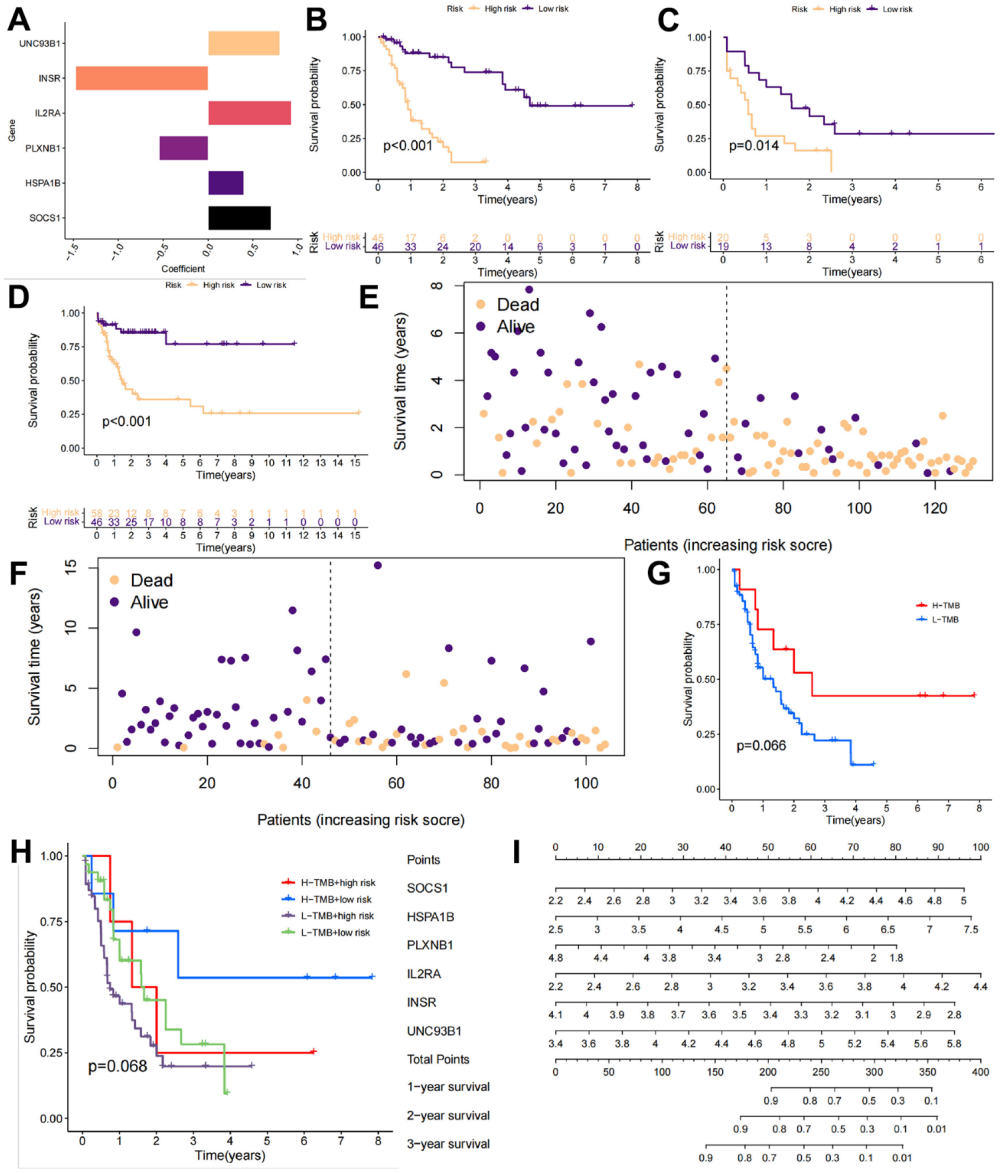
**Validation of the AML subtype training model.** (**A**) The GSE71014 cohort from the GEO database was downloaded as the internal test group, and the following final model coefficients were constructed: risk score = [value of SOCS1×(0.70)] + [value of HSPA1B×(0.40)] + [value of PLXNB1×(-0.55)] + [value of IL2RA×(0.93)] + [value of INSR×(-1.47)] + [value of UNC93B1×(0.80)]. (**B**–**D**) The training cohort, internal test group, and external test cohort were divided into high-risk and low-risk groups. (**E**, **F**) The risk scores and survival times of the different patients in the TCGA-LAML cohort and GSE71014 cohort. (**G**, **H**) The prognosis of the high-load patients was better than that of the low-load patients due to the presence of more mutation sites. (**I**) We constructed a column chart to better guide the identification of high-risk patients in the clinic.

The next objective was to identify a specific target for a candidate mRNA vaccine so that the prognosis of injected populations may be improved to a level consistent with that of the C2 in the TMBRG subgroup. A one-way Cox regression analysis was used to screen the TMBRGs for prognostic genes at p < 0.05. Multivariate Cox regression was used to analyze the results. Subsequently, a multifactorial Cox regression model was used for modeling. A ratio of 8:2 was used between the training and internal test groups in the TCGA-LAML cohort. Additionally, the GSE71014 cohort from the GEO database was classified as an external test group. The final model coefficients were constructed as follows: risk score = [value of SOCS1×(0.70)] + [value of HSPA1B×(0.40)] + [value of PLXNB1×(-0.55)] + [value of IL2RA×(0.93)] + [value of INSR×(-1.47)] + [value of UNC93B1×(0.80)] (Figure 5A).

We conducted broader research on the six genes we screened and found existing studies supporting their potential in tumor immunotherapy. Previous research has focused on aspects such as immunity and cell differentiation to explain why these genes have therapeutic potential. However, the premise for the immune system to eliminate cancer is its ability to induce programmed cell death. Interestingly, previous studies on AML have lacked information related to the apoptotic pathway. Therefore, we conducted analyses related to ferroptosis. Interestingly, the results we obtained coincided with those of previous research, mutually validating our findings.

There is a close relationship between UNC93B1, IL2RA, HSPA1B, and SOCS1 overexpression and chemotherapy resistance in patients with acute myeloid leukemia (AML). Specifically, UNC93B1, a transmembrane protein involved in regulating intracellular Toll-like receptor (TLR) signaling, plays a crucial role in this process. Its expression is significantly positively correlated with that of CD14, CD68, and almost all Toll-like receptors. The high expression of UNC93B1 is also associated with the infiltration of innate immune cells. This finding suggests a critical role for UNC93B1 in AML progression. Similarly, the α chain of IL-2RA encodes the IL-2 receptor, whose overexpression is closely associated with chemoresistance and poor prognosis in AML, and it has been shown that IL-2RA antibodies target leukemic cells without affecting normal hematopoietic cells. These findings show that this target has the potential for treating cancer and has a considerable degree of safety. HSPA1B is rarely reported in AML, but it has been shown to correlate with the progression of microvascular complications, suggesting its importance in tumorigenesis and tumor immunity. SOCS 1 has been identified as a negative regulator of the JAK/STAT pathway in several studies. Abnormal SOCS 1 expression not only promotes cell carcinogenesis but also controls the proliferation and differentiation of hematopoietic precursor cells via methylation of the SOCS 1 gene in AML patients, subsequently promoting the growth and proliferation of AML cells.

In contrast, INSR and PLXNB1 are protective genes and are downregulated upon AML relapse. The expression of INSR, an evolutionarily conserved signaling protein that is downregulated upon relapse in AML patients, may be related to the inhibition of tumor cell growth and proliferation. As a nodal gene, INSR was found to be downregulated at relapse by rule modeling in adult AML patients. There are few reports on the role of PLXNB1 in AML, but it plays an important role in the chondrogenic differentiation of bone marrow mesenchymal stem cells (BMSCs) and is regulated by miR-362-5p. High expression of PLXNB1 promotes the chondrogenic differentiation of BMSCs, and its downregulation upon AML relapse may have a protective effect against disease progression. The changes in the expression of these two genes provide new perspectives on the prognosis and treatment of AML patients, suggesting their potential value in individualized treatment.

The TRAINING cohort, internal test group, and external test group were divided into high-risk and low-risk groups according to the calculated risk score (Figure 5B–5D). In all three groups, the high-risk group had a significantly lower survival rate than the low-risk group, and this difference was statistically significant (p < 0.05). The risk scores and survival statuses of the different patients in the TCGA-LAML cohort and GSE71014 cohort are further shown in Figure 5E, 5F. Interestingly, we also compared the differences in survival between patients with a high TMB and those with high-risk scores. Due to the presence of more mutation sites, the prognosis of high-load patients was better than that of low-load patients (Figure 5G). We further categorized the patients into 4 groups according to TMB and risk score: H-TMB+high risk, H-TMB+low risk, L-TMB+high risk, and L-TMB+low risk. Low-risk scores and high TMB were associated with the best prognosis, while high-risk scores and low TMB were associated with a worse outcome. Considering the small sample size of the cohort, this may be the reason why the distinction did not reach the threshold of p < 0.05 after assessing the 4 groups. To facilitate the use of this model, we constructed a column-line diagram to better guide the identification of high-risk patients in the clinic (Figure 5I).

### Immune characteristics of the AML subtypes

**Figure 6:**
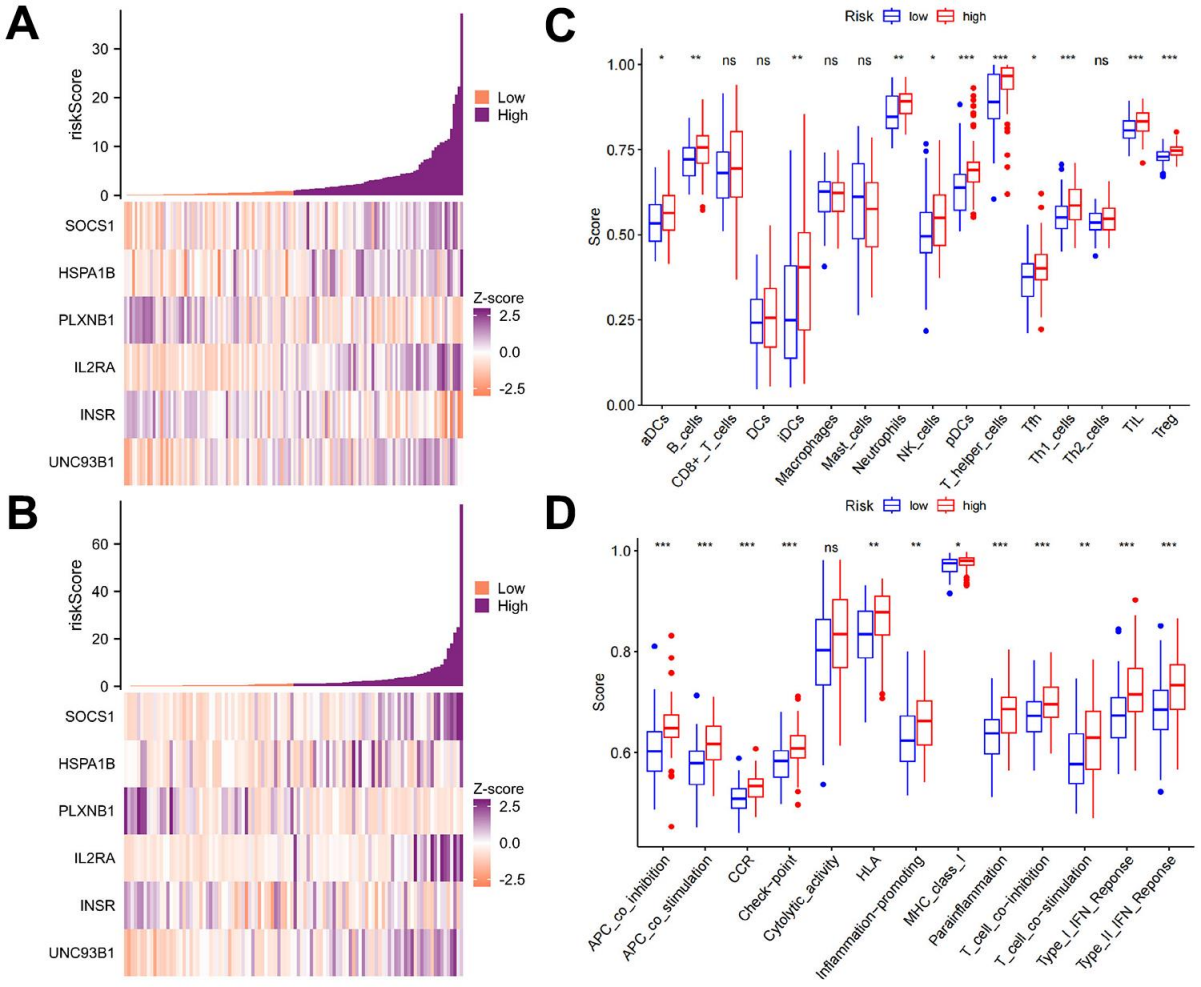
**Immune characteristics of the AML subtypes.** (**A**, **B**) The expression levels of the six genes in the TCGA-LAML and GSE71014 cohorts based on the ssGSEA calculations. (**C**, **D**) The differences in immune cells and immune functions between the high- and low-risk groups revealed that the high-risk group had more immune cells than the low-risk group for most of the immune cell types. Only two types of immune cells, macrophages, and mast cells, were more common in the low-risk group than in the high-risk group. The high-risk group was found to have greater immune function than the low-risk group.

We further examined the immune differences between patients with different risk scores. The first step was to examine the expression of the 6 genes in the TCGA-LAML and GSE71014 cohorts, as shown in Figure 6A, 6B. Based on our ssGSEA calculations, we examined immune cell differences and immune function differences between the high- and low-risk groups and found that the high-risk group had higher levels of aDCs, B cells, CD8+ T cells, DCs, iDCs, neutrophils, NK cells, pDCs, T-helper cells, Tfhs, Th1 cells, Th2 cells, TILs, and Tregs than did the low-risk group. Only two immune cell types, macrophages, and mast cells, were more common in the low-risk group than in the high-risk group (Figure 6C). The high-risk group showed higher immune function indicators than the low-risk group (Figure 6D). Thus, the high expression of the above six genes improved patient prognosis, and these genes could be candidates for mRNA vaccines in further research.

## DISCUSSION

We systematically examined the tumor mutation types in the TCGA-LAML cohort and investigated in depth the genetic variants of each patient in the TCGA-LAML cohort, focusing on two common variant types, missense mutations and single-nucleotide polymorphisms (SNPs). As shown in Figure 2A–2E, in the TCGA-LAML cohort, a missense mutation was the most common variant classification, while a SNP was the most common variant type. These findings highlight the important role of these mutations in the development of AML. In addition, we examined the detailed mutation profiles of the top 30 genes, as demonstrated in Figure 2F, 2G. These genes were selected based on their importance in the development of AML. We focused on the mutation frequencies and patterns of these genes to explore their biological significance in the disease process. This detailed mutation information provides a solid foundation for subsequent differential expression analysis and screening of candidate genes. Our goal was to identify genes closely associated with AML development, especially those related to immune function and ferroptosis regulation.

We performed differential gene analysis of the TCGA-LAML cohort by using the Limma package to clarify the changes in the expression of genes closely associated with AML development. To enrich our list of candidate genes and ensure that our subsequent studies covered key genes related to the regulation of immune function and ferroptosis in AML, we integrated information from ImmPort Shared Data and FerrDb V2. By taking the intersection, we identified a final list of 856 genes as candidate genes for subsequent analysis. Drug resistance remains a major challenge in cancer treatment. Interestingly, a ferroptosis-induced reversal of cancer resistance has been observed when ferroptosis is induced [29]. Moreover, Tumor research relies heavily on TMB [30, 31]; as a result, it is crucial to investigate TMB’s association with immune and ferroptosis genes. We examined the TMB accumulation of each patient in the TCGA-LAML cohort and defined the highest one-third of patients as “high mutation load patients” and the lowest one-third as “low mutation load patients” for further analysis. Through the application of WGCNA, we identified the immune- and ferroptosis-related genes associated with TMB and divided the network into six coexpression modules by dynamic tree cutting; these modules contained different numbers of genes of different colors. In the ensuing association analysis, we specifically emphasized the relationship between TMBRGs and these modules and explored the association of each module with TMB at the p < 0.05 significance level. During this step, immune and ferroptosis genes associated with TMB were identified and their expression patterns at different TMB levels were examined.

Due to its heterogeneity, AML has a poor prognosis and a low rate of recurrence. Tumor cells are aggressive and sensitive to treatment based on their molecular and genetic characteristics [32]. Thus, integrating subtypes into the clinical management of AML is crucial [33]. We screened TMBRGs by univariate Cox regression analysis and constructed a prediction model to classify patients into two distinct subgroups, C1 and C2, to further investigate the association between TMB and patient survival. First, we performed a one-way Cox regression analysis of the TMBRGs and used p < 0.01 as the threshold for screening to identify genes associated with patient prognosis. The purpose of this step was to construct a more accurate prediction model to ensure that the selected genes were significantly associated with patient survival. Survival analysis based on these TMBRGs showed that we successfully categorized the patients into two distinct groups—C1 and C2—and that the survival of the C2 patients was significantly better than that of the C1 patients. This difference was demonstrated in the survival curves, p < 0.001, which strongly emphasized the significant difference in survival between these two subgroups. These findings suggest that TMBRGs play a key role in stratifying the survival risk of patients, providing important clues for further individualized treatment planning, which is also in line with previous findings [34]. The survival advantage of C2 patients may reflect the positive response of their tumor immune environment, which is related to the synergistic effect of TMB [35].

Several studies have demonstrated that the tumor microenvironment significantly influences treatment response and clinical outcomes [36, 37]. We used the ESTIMATE algorithm and the MCP-counter algorithm to conduct an in-depth analysis of the tumor microenvironment of the patients, with special attention given to the differences between the C1 and C2 patients, to explore the immune characteristics of these two subgroups. First, there was an overall difference in the tumor microenvironment between C1 and C2 patients based on the ESTIMATE algorithm. Compared to C2 patients, C1 patients scored higher on stromal, immune, and ESTIMATE scores, implying that the tumor microenvironment of the C1 patients was relatively greater in terms of the tumor stroma and immune cell penetration. In the C1 and C2 patients, there was a significant difference in the overall composition of the tumor microenvironment, which may have a profound impact on patient treatment response and prognosis. Using the MCP-counter algorithm, we quantified the immune and stromal cell abundance. Among the eight immune cell types and two stromal cell types, the abundance of three immune cell types—monocytic lineage cells, myeloid dendritic cells, and neutrophils—was significantly higher in C1 patients compared to C2 patients. This difference in immune cells may be consistent with the relatively higher stromal and immune scores in the C1 patients, further highlighting the significant difference in immune cell composition between the C1 and C2 patients. The significant increase in immune cells in the C1 patients may reflect a stronger immune response to tumor antigens generated by TMBRGs, which provides valuable clues for vaccine target research and development. Fully understanding the difference between the C1 and C2 patients at the immune cell level helps us to more accurately predict a patient’s response to immunotherapy and provides more information for individualized treatment planning.

We ultimately screened six candidate genes, UNC93B1, INSR, IL2RA, PLXNB1, HSPA1B, and SOCS1. Although these candidate genes require functional validation, previous reports support their potential in tumor immunotherapy. For instance, IL2RA encodes the alpha chain of the interleukin-2 receptor and represents a low-affinity receptor for interleukin-2 (IL-2). Along with IL2RB (CD122) and IL2RG (CD133), these proteins form the high-affinity IL-2 receptor [38, 39]. Overexpression of IL2RA, encoding the alpha chain of the IL2 receptor, is linked to chemotherapy resistance and poor outcomes in AML patients. IL2RA antibodies inhibit leukemic but not normal hematopoietic cells [40–42]. SOCS1 is a member of the SOCS protein family. SOCS1 functions as a negative regulator of the JAK/STAT pathway, suppressing intracellular signal transduction activated by cytokines. This regulatory role is crucial in controlling the proliferation and differentiation of hematopoietic precursor cells. Aberrant expression of SOCS1 promotes cell transformation and contributes to carcinogenesis [43–45]. The INSR gene encodes a highly conserved signaling protein that plays diverse roles in metazoan development. Furthermore, INSR is downregulated at relapse in adult AML patients via rule-based modeling, where it serves as a node gene [46, 47]. Furthermore, high UNC93B1 expression tends to be associated with the infiltration of innate immune cells, including macrophages, dendritic cells, neutrophils, eosinophils, and NK CD56dim cells [48]. Therefore, the UNC93B1 gene plays a critical role in the advancement of AML.

Unlike the IL2RA, SOCS1, INSR, and UNC93B1 genes, the PLXNB1 and HSPA1B genes have been less commonly reported in AML-related studies. PLXNB1 exhibits high expression during BMSCs’ chondrogenic differentiation, and its overexpression enhances this process. Mechanistically, PLXNB1 is a target of miR-362-5p [49]. The HSPA1B gene has long been recognized to be involved in microvascular complication progression. One possible reason for the relatively small number of reports on the HSPA1B gene in AML is that HSPA1B is associated with most microvascular complications [50], and microvascular complications are one of the links in tumorigenesis. This finding suggests a key role for the HSPA1B gene in follow-up studies of tumor immunity.

In our study, we analyzed the close relationship between TMBRGs and patient prognosis and explored the potential value of these genes as potential vaccine targets. First, we screened for TMBRGs by Cox regression analysis and constructed a reliable prediction model to classify patients into high- and low-risk groups. This model demonstrated strong predictive performance in both the TCGA-LAML cohort and GSE71014 cohort. Patients in the high-risk group exhibited significantly lower survival rates compared to those in the low-risk group, a statistically significant difference observed across cohorts and subgroups. This provides strong support for the robustness and utility of our model. Furthermore, we compared the survival of high-load and high-risk patients. In line with our expectation, the survival of patients with high tumor loads was relatively better because they had more mutation sites. This result reinforces the importance of TMB in predicting patient prognosis and provides a biological rationale for the use of TMBRGs as potential vaccine targets.

We explored the association of TMBRGs with the immune status of patients and focused on the potential role of these genes in immunotherapy and their potential as vaccine targets. First, we conducted a detailed investigation into the differences in immune cells and immune functions between the high-risk and low-risk groups using ssGSEA-based calculations. Our findings revealed that elevated expression of TMBRGs correlated with enhanced immune activity, underscoring the critical role of these genes in influencing patients’ immune status. This heightened immune cell activity may serve as a biological foundation for the poorer prognosis observed in the high-risk group. Second, we analyzed the association between high TMBRG expression and better prognosis. By comparing the survival of the high-load and high-risk patients, we found that the patients with high expressions of TMBRGs had a better prognosis. This result further emphasizes the positive role of these genes in tumor biology and provides a biological rationale for their use as potential vaccine targets. Finally, we investigated whether high TMBRG expression could be a potential vaccine target. Through an in-depth analysis of the genes identified in our study, we suggest that these genes may be important targets for tumor immunotherapy. The association of these genes with immune differences suggested that regulating the expression of these genes may affect the activity of immune cells and thus have a positive effect on tumors. Therefore, the high expression of TMBRG is potentially valuable as a vaccine target [51, 52] and deserves additional in-depth experimental studies and clinical validation in the future.

The novelty of this study lies in the comprehensive analysis of tumor mutational burden (TMB) and gene mutations in acute myeloid leukemia (AML) patients by systematically integrating various bioinformatics tools and databases. Using TCGA-LAML cohort data, we identified the most common mutation types in AML, including missense mutations and SNPs, and further analyzed the distribution and biological significance of the 30 most frequently mutated genes. Additionally, by integrating the ImmPort Shared Data and FerrDb V2 databases, we identified 856 candidate genes related to immunity and ferroptosis and used weighted gene coexpression network analysis (WGCNA) to identify gene modules associated with TMB. We developed a predictive model based on TMB-related genes to classify AML patients into high-risk and low-risk groups, and we validated the model’s efficacy. Finally, using the ssGSEA and ESTIMATE algorithms, we conducted a thorough analysis of the tumor microenvironment and immune cell variations among patients. This analysis identifies novel potential targets for mRNA vaccine design. This comprehensive multilevel analysis approach is unprecedented in AML research and provides an important theoretical foundation for personalized treatment.

Despite the novel findings of this study, there are limitations. Due to the unique nature of AML as a hematologic malignancy, the difficulty in sample collection is significantly greater than that of common solid tumors. This results in generally smaller sample sizes in bioinformatics studies of hematologic malignancies, including ours. This may affect the robustness and generalizability of the results, necessitating further validation in subsequent clinical studies. Future research can expand on this study in several dimensions. For example, other biomarkers, such as proteomics and metabolomics, could be integrated for multidimensional comprehensive analysis to further elucidate the complex pathogenesis of AML. Additionally, in vaccine development, based on the findings of this study, further exploration of the specific identification and functional research of mRNA vaccine targets is needed to expedite the translation of research findings into clinical applications.

## MATERIALS AND METHODS

### Acute myeloid leukemia dataset source and preprocessing

The workflow of our study is shown in Figure 1. A total of 151 LAML patients were identified in the TCGA database, and their data included RNA-seq (FPKM) data, variant VarScan data, and clinical information. We excluded patients without survival information. For the control group, we obtained sequence data from the GTEx database. To mitigate batch effects arising from nonbiological technical biases, we applied the “ComBat” algorithm from the sva package and conducted differential gene analysis. Additionally, we utilized the GSE71014 cohort from the GEO database as an external validation dataset.

### Comparison of genetic alterations

The cBioCancer Genomics Portal (cBioPortal, https://www.cbioportal.org/) was used to integrate the raw RNA-seq data from the TCGA and other databases and compare genetic alterations in AML patients. P-values < 0.05 were considered to indicate statistical significance.

### WGCNA analyses

To explore immune- and ferroptosis-related genes associated with TMB, we conducted weighted gene coexpression network analysis (WGCNA) to construct coexpression modules of differentially expressed genes (DEGs). Using the WGCNA R package (v1.68), we identified coexpressed modules that represented diverse subtypes in the TCGA cohort. These modules were clustered to observe similarities among them. Module significance indicated the relationship between modules and TMB status. Module membership, defined as the correlation coefficient between genes and module eigengenes, assessed the reliability of genes within each module. Gene significance reflected the association between genes and traits. Significant modules were identified based on their correlation with TMB-related characteristics, and core genes from the most correlated module were identified as TMB-associated genes. Subsequently, we used the ClusterProfiler R package (v3.14.3) to perform Gene Ontology (GO) and Kyoto Encyclopedia of Genes and Genomes (KEGG) analyses of these TMB-associated genes.

### Identification and validation of AML subtypes

Genes from both the TCGA-LAML and GTEx cohorts were selected to delineate distinct ferroptosis regulation patterns mediated by ferroptosis-related genes (FRGs). Employing unsupervised clustering analysis, we aimed to identify these patterns based on gene expression and classify patients for further investigation. The number and stability of clusters were determined using the consensus clustering algorithm implemented via the “ConsensusClusterPlus” package, ensuring robust classification through 1000 repetitions. Subsequently, the TCGA-LAML cohort was partitioned into a training group and an internal test group at an 8:2 ratio. We validated the results of unsupervised clustering in the internal test group. Principal component analysis (PCA), a method frequently used for dimensionality reduction, was employed to confirm the reliability of the consensus clusters.

### Estimation

The efficacy of vaccines is intricately linked to the tumor immune microenvironment. We used the ESTIMATE algorithm to perform an in-depth tumor microenvironment analysis of the patients in the LAML cohort to explore its predictive role in vaccine therapy. In particular, we focused on the significant differences in the tumor microenvironment between the C1 and C2 patients. The ESTIMATE algorithm provided comprehensive information on the tumor microenvironment by evaluating the relative proportions of immune and nonimmune cells in tumor tissue. Using this algorithm, we derived stromal scores, immune scores, and estimated scores to assess the relative abundance of stromal and immune cells within the patients’ tumor tissues. Subsequent analysis uncovered notable disparities in the tumor microenvironment between the C1 and C2 groups.

### Kaplan-Meier survival analysis

According to the median value of the risk score (calculated using the “survminer” R package), AML patients were categorized into high-risk and low-risk groups. Kaplan-Meier survival analysis was conducted to evaluate the survival rate and median survival time for each group. The log-rank test was employed to assess differences in survival between these groups. Furthermore, a time-dependent receiver operating characteristic (ROC) curve was generated using the “time ROC” R package to determine the specificity and sensitivity of the risk model.

### ssGSEA

Normalized TCGA GTEx data were juxtaposed with the gene set utilizing the “GSVA” algorithm (R package). ssGSEA categorizes gene sets based on shared biological functionalities, chromosomal localization, and physiological regulation [53]. We identified 16 types of immune cells: activated dendritic cells (aDCs), B cells, CD8+ T cells, dendritic cells (DCs), immature dendritic cells (iDCs), neutrophils, NK cells, plasmacytoid dendritic cells (pDCs), T-helper cells, follicular T-helper cells (Tfhs), Th1 cells, Th2 cells, tumor-infiltrating lymphocytes (TILs), regulatory T cells (Tregs), macrophages, and mast cells. Additionally, 13 types of immune functions were categorized: antigen-presenting cell (APC) coinhibition, APC costimulation, chemokine receptor (CCR), checkpoint cytolytic activity, human leukocyte antigen (HLA), inflammation-promoting major histocompatibility complex (MHC) class I, parainflammation, T-cell coinhibition, T-cell costimulation, type I interferon (IFN) response, and type II IFN response.

Using the ssGSEA scores of each sample, we conducted unsupervised classification of PDAC samples using the ConsensusClusterPlus R package (v1.50.0) with a consensus clustering algorithm. Principal component analysis (PCA) was employed to evaluate the clustering effect of the model.

### Statistical analysis

All statistical analyses were conducted using R (version 3.6.1). The Wilcoxon test was utilized to assess differences between two groups, while ANOVA was employed for comparisons involving multiple groups. A significance level of p < 0.05 was deemed indicative of statistical significance.
